# The Impact of a Pure Protein Load on the Glucose Levels in Type 1 Diabetes Patients Treated with Insulin Pumps

**DOI:** 10.1155/2015/216918

**Published:** 2015-02-12

**Authors:** Tomasz Klupa, Teresa Benbenek-Klupa, Bartlomiej Matejko, Sandra Mrozinska, Maciej T. Malecki

**Affiliations:** ^1^Department of Metabolic Diseases, Jagiellonian University Medical College, Krakow, Poland; ^2^University Hospital, Krakow, Poland; ^3^DiabWay Enterprise, Krakow, Poland

## Abstract

We aimed to estimate the impact of ingestion of a pure protein load on the glucose levels in T1DM patients treated with insulin pumps. We examined 10 T1DM patients (6 females, mean age—32.3 years, mean HbA1c—6.8%) treated with insulin pumps equipped with a continuous glucose monitoring system (CGMS). In Phase I, baseline insulin infusion was optimized to minimize the differences in fasting glucose levels to less than 30 mg/dL between any two time points between 9 a.m. and 3 p.m. In Phase II, the patients were exposed to single pure protein load. CGMS record was performed and the glucose pattern was defined for 6 hours of each phase. The maximal glucose level increment was similar for the entire duration of the fasting and the protein load test (26.6 versus 27.6 mg/dL, resp., *P* < 0.78). There was only a borderline difference in change between baseline versus 6th hour glucose (12.5 and 19.0 mg/dL, *P* = 0.04). Glucose variability, assessed by standard deviation of mean glucose levels, was 36.4 and 37.9 mg/dL, respectively (*P* = 0.01). The administration of a pure protein load does not seem to have a clinically significant impact on glucose levels in T1DM patients treated with insulin pumps.

## 1. Introduction

Nutrition therapy is essential for the management of diabetes [1]. It is well recognized that the mealtime insulin requirement in type 1 diabetes (T1DM) patients is driven mostly by carbohydrate content and that monitoring it can improve glucose levels. The American Diabetes Association recommends meal plans based on carbohydrate counting as a key strategy to achieve glycemic control [2]. An impact of protein containing food on glycemia in diabetes has been studied for many decades; however, it is unclear whether or not the calculation of other food components can also be used to optimize glucose levels [3, 4]. Some studies have suggested that counting the amount of fat and protein (using fat/protein exchanges) may be beneficial for reaching glycemic control in T1DM children [5]. Such an approach could be particularly feasible in T1DM patients treated with continuous subcutaneous insulin infusion (CSII) via insulin pump [3]. One of the features implemented in some personal insulin pumps is the dual-wave bolus (DWB) option, which delivers the combination of an instant standard premeal insulin bolus followed by a square bolus (SB) infused over several hours, helping to tailor prandial insulin delivery to the composition of a meal [6]. This pump option may be particularly useful for mixed, fat-/protein-rich meals since such food seems to modify postmeal glucose patterns with a less rapid and more prolonged increase in plasma glucose concentration [7]. It has recently been shown among a cohort of pediatric patients with long-lasting T1DM, using pump therapy, that for mixed meals insulin dosing based on both carbohydrate and fat/protein counting leads to lower postprandial glycemic levels than the conventional counting of carbohydrates only. [8]. However, the studies performed so far estimating the glycemic effect, mostly on postprandial glucose levels, of fat and protein ingestion had some serious limitations. For example, they evaluated either the effect of a meal containing both fat and protein [5, 8] or the effect of just fat added to a mixed meal [9]. To our knowledge, until now no study has examined the effect of sole protein ingestion.

The aim of this study was to estimate the impact of ingestion of a pure protein load on glucose levels in T1DM patients treated with personal insulin pumps.

## 2. Methods

We examined 10 T1DM patients (6 females, 4 males) treated with insulin pumps (Medtronic Paradigm 722 or Veo) equipped with a continuous glucose monitoring system (CGMS) option. During the study, Enlite sensors (Medtronic) were used exclusively. All of the patients were white Caucasians under the medical care of the Department of Metabolic Diseases, University Hospital, Krakow, Poland. The selection of patients was based on good prestudy compliance as assessed by good glycemic control (HbA1c level less than 7.5%, 58 mmol/mol), the patient's usage of the Bolus Wizard (bolus calculator) option for more than 90% of boluses, changing infusion sets according to manufacturer's recommendation, and using at least one electrode for CGMS per month during the preceding year. The study participants were free from advanced chronic complications of diabetes. All patients gave informed consent, and the study was approved by the Bioethical Committee of the Jagiellonian University.

Before study entry (one to two weeks preceding Day 1), the patients' baseline insulin infusion was optimized to minimize the differences in fasting glucose levels to less than 30 mg/dL between any two time points between 9 a.m. and 3 p.m. The procedure of optimization was based on a retrospective analysis of CGMS records and involved increasing or decreasing the rate of basal insulin infusion two hours before the observed rise or fall in glucose level. Subsequently, the new settings of the basal infusion rate were rechecked with CGMS to meet the study criteria. In Phase I (Day 1), a fasting test (between 9 a.m. and 3 p.m.) was performed to confirm the stable glucose patterns (as defined above) on current basal insulin infusion settings.

In Phase II (Day 2), the patients were exposed to a single pure protein load by ingesting a commercially available preparation (Protifar, Nutricia, milk derived proteins). In its 100 mL volume, the product contained 88.5 grams of pure protein, 1.6 grams of fat, and less than 1.5 grams of carbohydrates (lactose), as well as minerals and micronutrients. All the patients were exposed to a dose of 0.3 g of pure protein (0.34 mL of Protifar/kg dissolved in 200 mL of water) for each kg of body weight; the load was administered at 9 a.m. Such a dose of protein is the equivalent of the usual protein portion in a medium-size meal, based on the dietary recommendations for patients with diabetes (15–20% of total daily energy intake) [2]. Both phases were performed in home settings; the patients were given precise instructions concerning the procedure.

The rate of basal insulin infusion during the protein load test was the same as that defined initially in Phase I; no modifications or extra insulin boluses were permitted. CGMS record was performed in both phases, during which the patients avoided physical activity. Glucose patterns were recorded during 6 hours of Phase I (fasting) and 6 hours of Phase II (protein load).

There was no consistency in the age of CGM sensors during Phase I and Phase II; however, we strictly avoided performing tests during the first or last (6th) day after sensor insertion.

The statistical analysis was performed using STATISTICA 10.0 software. The difference between the two phases was assessed by using the dependent* t*-test or paired sample Wilcoxon signed rank test, when applicable.

## 3. Results

**Figure 1:**
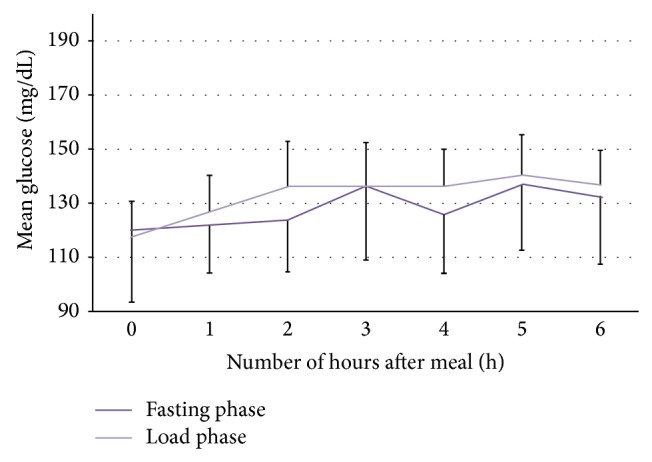
Glucose patterns in the study subjects during fasting state and after protein load, for the same rate of basal insulin dose. Data are presented as mean glucose and standard deviation.

The patients' mean age was 32.3 years, mean T1DM duration was 11.7 years, and mean HbA1c was 6.85% (51 mmol/mol). The clinical characteristics of the study participants are presented in Table 1. Mean baseline glucose levels were 119.8 and 117.6 mg/dL for Phase I and Phase II, respectively (*P* = 0.68). Mean maximal glucose levels were 146.4 and 145.2 mg/dL for Phase I and Phase II, respectively (*P* = 0.85). Mean maximal glucose level increment was similar for the entire 6-hour fasting and protein load test (26.6 mg/dL versus 27.6 mg/dL, resp., *P* = 0.78) (Figure 1). There was only a borderline difference between the change in baseline versus 6th hour glucose levels for the fasting state versus protein load test (12.5 mg/dL and 19.0 mg/dL, resp., *P* = 0.04). The glucose variability assessed by CGMS-based standard deviation of mean glucose levels was 36.4 and 38.9 mg/dL for Phase I and Phase II, respectively (*P* = 0.01).

There were no episodes of infusion set malfunctions during Phase I or Phase II of the study, and no hypoglycemia was recorded during fasting or after loading.

## 4. Discussion

Here, for the first time, we evaluated the impact of the ingestion of a pure protein load on glucose levels in adult T1DM subjects treated with insulin pumps. In this short-term clinical experiment, we found that protein consumption had very little effect on the glycemia of the examined cohort of patients.

Contradictory to our study results, there have been several earlier papers reporting a substantial rise in glucose levels in T1DM patients as an effect of the consumption of noncarbohydrate food components [5, 8, 10]. However, these reports involved either fat only or fat and protein combined, because, in real-life settings, they are often consumed together in products chosen by diabetic individuals, such as meat, poultry, fish, and dairy products. Thus, the discrepancy between earlier studies and our results may be explained by the variable short-term impact of fat and protein versus protein only consumption on glucose homeostasis through different mechanisms of hepatic gluconeogenesis, postmeal insulin resistance, release of glucagon and gut hormones, gastric emptying, and rate of absorption of other food components like carbohydrates and other factors [11–13]. There are some earlier reports from different healthy and diabetic populations suggesting variable glycemic effects of high protein meals; for example, they promote gluconeogenesis and slow gastric emptying [13, 14]. In a recently published study in T1DM, it was shown that a mixed meal consisting of 30 g of carbohydrate and 40 g of protein has a greater glycemic effect than a pure carbohydrate load [10]. This seems to be in conflict with the results of our analysis. However, there were substantial differences between the two studies. The study by Smart was performed in a pediatric population, while ours involved adult individuals. The differences in outcomes of both studies may be associated with various physiological reactions to protein load in the examined age groups. Furthermore, there may be discrepancies in the proportion of basal versus prandial insulin doses depending on the age of patients, which could affect study outcomes. Moreover, the effect of protein when mixed with carbohydrates might be different compared to the effect of protein alone. Finally, the impact of protein may be dose-dependent, because, in the earlier study, 40 g of protein per meal was used, while the protein dose in our study was much lower.

It is also important to underline that factors other than meal size or composition may influence the rate of food absorption and postprandial glucose patterns. The list of such factors includes the method of preparation (e.g., cooking, frying, or grilling), rapidity of consumption, accompanying beverages, time elapsed since the previous meal (second-meal phenomenon), and variability in individual rates of gut absorption, as well as some other factors [15]. Moreover, various types of protein consumed as a meal component or during the load test might have affected the postmeal/load glucose patterns [16]. All these factors could have been responsible for the differences between the studies.

The outcomes of our study may have practical clinical consequences as there are some small protein-based snacks available, such as protein bars or shakes, which are small meal options for T1DM patients. So, it is important to provide them with information about their impact on glucose level. This is particularly important as, through their satiating effect, they may be helpful in weight management [17]. An important advantage of our study was the carefully optimized rate of basal insulin infusion based on the earlier fasting phase of the experiment. Consequently, we were able to exclude the potential bias related to an excess or deficit of basal insulin on postload glucose patterns.

A shortcoming of this report is the small number of examined T1DM individuals. Thus, an independent confirmation based on a larger population is required. Additionally, our subjects were highly preselected; they were adult T1DM patients characterized by very good compliance prior to the study, frequent CGMS use, and willingness to follow a challenging study protocol. Consequently, caution is required when extrapolating to other diabetic populations. The direct clinical implication of our study is the recommendation that small protein-based snacks may be consumed by adult T1DM CSII-treated patients without insulin bolusing. Obviously, the general guidelines concerning daily protein consumption for patients with diabetes should be followed [2]. The results of this study cannot be generalized to other noncarbohydrate food components, especially fat. Additionally, we examined glucose patterns for 6 hours; thus, it is not possible to exclude the protein load effect on glycemia beyond this time, for example, during subsequent meal. Finally one has to underline, that we have used CGM systems that are still characterized with limited accuracy and reliability [18].

## 5. Summary

In conclusion, the ingestion of a pure protein load does not seem to have a clinically significant impact on glucose levels in adult T1DM patients treated with insulin pumps. Thus, small protein-based snacks do not require prandial insulin bolusing.

## Figures and Tables

**Table 1:** Clinical characteristics of study participants.

Variable	Whole study group		
	Mean	SD	Median
Gender F/M [*n*/*n*]	6/4	—	—
HbA1c [% | mmol/mol]	6.8 | 51	0.4 | 4	6.9 | 52
Age at time of examination [yrs]	32.3	8.6	30.5
T1DM duration [yrs]	11.7	6.4	9.5
Weight [kg]	71.1	12.7	70.6
Average daily insulin dose per kg of body mass [IU/kg]	0.53	0.06	0.54
Average percentage of basal insulin [%]	40.9	3.4	40.5
